# Ligand Recognition of the Major Birch Pollen Allergen Bet v 1 is Isoform Dependent

**DOI:** 10.1371/journal.pone.0128677

**Published:** 2015-06-04

**Authors:** Christian Seutter von Loetzen, Thessa Jacob, Olivia Hartl-Spiegelhauer, Lothar Vogel, Dirk Schiller, Cornelia Spörlein-Güttler, Rainer Schobert, Stefan Vieths, Maximilian Johannes Hartl, Paul Rösch

**Affiliations:** 1 Department of Biopolymers, University of Bayreuth, Bayreuth, Bavaria, Germany; 2 Chair of Organic Chemistry, University of Bayreuth, Bayreuth, Bavaria, Germany; 3 Division of Allergology, Paul-Ehrlich-Institut, Langen, Hesse, Germany; Russian Academy of Sciences, Institute for Biological Instrumentation, RUSSIAN FEDERATION

## Abstract

Each spring millions of patients suffer from allergies when birch pollen is released into the air. In most cases, the major pollen allergen Bet v 1 is the elicitor of the allergy symptoms. Bet v 1 comes in a variety of isoforms that share virtually identical conformations, but their relative concentrations are plant-specific. Glycosylated flavonoids, such as quercetin-3-O-sophoroside, are the physiological ligands of Bet v 1, and here we found that three isoforms differing in their allergenic potential also show an individual, highly specific binding behaviour for the different ligands. This specificity is driven by the sugar moieties of the ligands rather than the flavonols. While the influence of the ligands on the allergenicity of the Bet v 1 isoforms may be limited, the isoform and ligand mixtures add up to a complex and thus individual fingerprint of the pollen. We suggest that this mixture is not only acting as an effective chemical sunscreen for pollen DNA, but may also play an important role in recognition processes during pollination.

## Introduction

Allergies are a major health problem worldwide. In particular, type I or immediate type allergies [1] that involve proteins as causative agents are very widespread and potentially severe. The major birch pollen allergen Bet v 1 from the European white birch (*Betula verrucosa*) alone [2] affects an estimated 100 million people [3]. Although birch pollen contain a variety of allergens from different protein families, more than 60% of all birch pollen-allergic patients react exclusively to Bet v 1 [4]. Up to 90% of the Bet v 1-sensitized patients also exhibit IgE-mediated allergic cross-reactions (oral allergy syndrome) to Bet v 1-homologous food allergens, with fruits, vegetables, and nuts as the most important elicitors of the allergy [5,6].

On the basis of sequence similarities and the protein three-dimensional structures, Bet v 1 and related pollen and food allergens belong to the family of class 10 pathogenesis-related proteins (PR-10) within the Bet v 1 superfamily. It was suggested that proteins in this family are involved in plant defense mechanisms, since expression of the respective genes is induced upon attacks of pathogens and by environmental stress [7]. However, the physiological roles of PR-10 proteins seem to extend beyond stress and pathogen response. Thus, the PR-10 strawberry allergen Fra a 1 is involved in controlling flavonoid biosynthesis and this protein is capable of binding different metabolic intermediates [8]. In general, PR-10 proteins often co-occur with flavonoids *in vivo* [9–15] and interact with flavonoids *in vitro* [8,16], as clearly evidenced, for example, for Bet v 1 [17,18]. Why many, if not all, PR-10 proteins appear as mixtures of isoforms, however, remains elusive [19–21].

The first Bet v 1 isoform described on the DNA level was Bet v 1a [22] followed by the identification of numerous other isoform sequences [23–25]. At least 18 Bet v 1 variants found in pollen on the mRNA or protein level [23,26,27] are officially listed as isoallergens (http://www.allergen.org). Studies on the proteomic profile of birch pollen extracts of different origin or species revealed significant differences of isoform composition and quantity [26,27]. For example, Bet v 1 constitutes up to 30% of the total protein content in Swedish pollen and 12% in Austrian pollen. In all cases so far, the most abundant isoform is Bet v 1a (50% to 70%), followed by Bet v 1d (20%), Bet v 1b (3% to 20%), Bet v 1f (2% to 8%), and Bet v 1j (~1%) [26].

Bet v 1a is well characterized by biochemical [2,18,28] and structural [29–31] studies. The large hydrophobic pocket formed by the secondary structure elements of Bet v 1 suggested that this allergen acts as storage or carrier protein [29,32,33]. Previous research work focused on trial-and-error approaches or docking simulations to test various ligands for binding to recombinant Bet v 1 [18,30,34]. We recently purified Bet v 1 in complex with its natural ligand quercetin-3-O-sophoroside (Q3OS) directly from mature birch pollen and confirmed binding by reconstitution of the Bet v 1a:Q3OS complex from its recombinant protein and synthetic ligand component [17]. We hypothesized that this complex may be involved in UV-protection of the pollen DNA and that Q3OS may stimulate pollen tube formation upon rehydration of the pollen. We then asked why different isoforms exist and whether there are physiological ligands other than Q3OS. Although it is tempting to believe on the basis of the high sequence identities of 87.4%–99.4% to Bet v 1a that all isoforms specifically interact with Q3OS, Bet v 1 isoforms are strikingly different in their immunological and allergenic properties [35] and, although allergenicity is mainly correlated with binding epitopes at the surface of allergens [36] it has always been speculated that Bet v 1 proteins as such are only part of the story, and that IgE binding needs to be tested in complex with their natural binding partners to arrive at meaningful results [30].

In order to characterize serological IgE binding as a measure for allergenicity as well as the physiological function of Bet v 1, we thoroughly studied ligand- and antibody-binding behaviour of the Bet v 1 isoforms a (hyperallergen), m (intermediate), and d (hypoallergen). Surprisingly, while none of the ligands significantly alters the allergenicity of Bet v 1, ligand binding to the different isoforms is diverse and highly dependent on the composition of the ligands’ sugar moieties.

## Results and Discussion

### Bet v 1:Q3OS interaction is isoform-dependent

**Fig 1:**
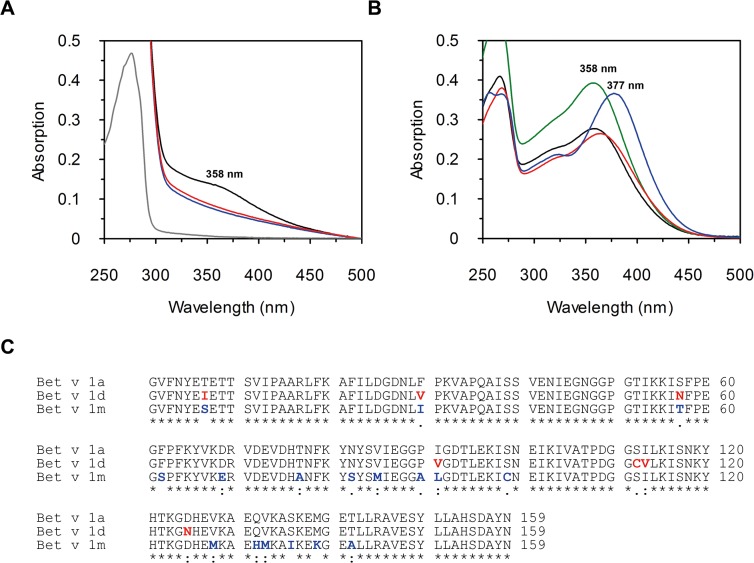
UV/VIS spectroscopy of flavonoids and Bet v 1 isoforms. All spectra were recorded at 298 K with 50 mM sodium phosphate, 50 mM NaCl at pH 7.0 and 10% DMSO as sample buffer. **A** Binding of Q3OS to Bet v 1 isoforms. UV/VIS spectra of 20 *μ*M Bet v 1a (**-**) and Q3OS incubated with Bet v 1a (-), Bet v 1d (-) and Bet v 1m (-) concentrated and subsequently eluted from a G25 column. **B** UV/VIS spectra of 20 *μ*M Q3OS (-), quercetin (-), Q3OGlc (-) and Q3OGal (-) reveal differences in absorption maxima and intensities. **C** Sequence alignment of the Bet v 1 isoforms a, d and m as performed with ClustalW [91]. Amino acids are marked with asterisks (identical), colons (conserved) and dots (semi-conserved). Residues that vary compared to Bet v 1a are highlighted in red for Bet v 1d (95.6% sequence identity to Bet v 1a) and in blue for Bet v 1m (89.3% sequence identity to Bet v 1a).

We were asking whether isoforms a, d, and m form identical complexes with the Bet v 1a natural ligand Q3OS [17]. In an initial experiment we noticed that Q3OS exhibits slightly different shades of yellow when incubated with these Bet v 1 isoforms. After incubation we removed excess Q3OS with a G25 column and recorded UV/VIS absorption spectra of the protein fractions (Fig 1A) and of unbound Q3OS (Fig 1B). In the presence of Bet v 1a, the UV/VIS spectrum of Q3OS shows a clear shoulder around 360 nm, while this is not the case for Bet v 1 isoforms d or m. These absorbance differences suggest that the putative Bet v 1d:Q3OS and Bet v 1m:Q3OS complexes are different from the Bet v 1a:Q3OS complex.

### Binding of unglycosylated flavonoids to Bet v 1 isoforms

Since the determination of the three-dimensional structure of Bet v 1a in 1996 [29] it has been suggested that the protein functions as a carrier or storage protein. The existence of various highly similar, structurally almost identical isoforms could be evidence for a complex network of different acceptors, targeted to bind chemically similar ligands. Hitherto, there is only limited comparable information available about differences in ligand binding behaviour between Bet v 1 isoforms of different allergenic potential. Recent approaches used indirect methods (ANS replacement assay, [18]) or analysed ligand binding in protein crystals [30,37]. We now used UV/VIS and NMR spectroscopy to systematically analyse and compare binding of physiologically relevant ligands to three different Bet v 1 isoforms (Fig 1C) in solution, with a focus on flavonoids.

**Fig 2:**
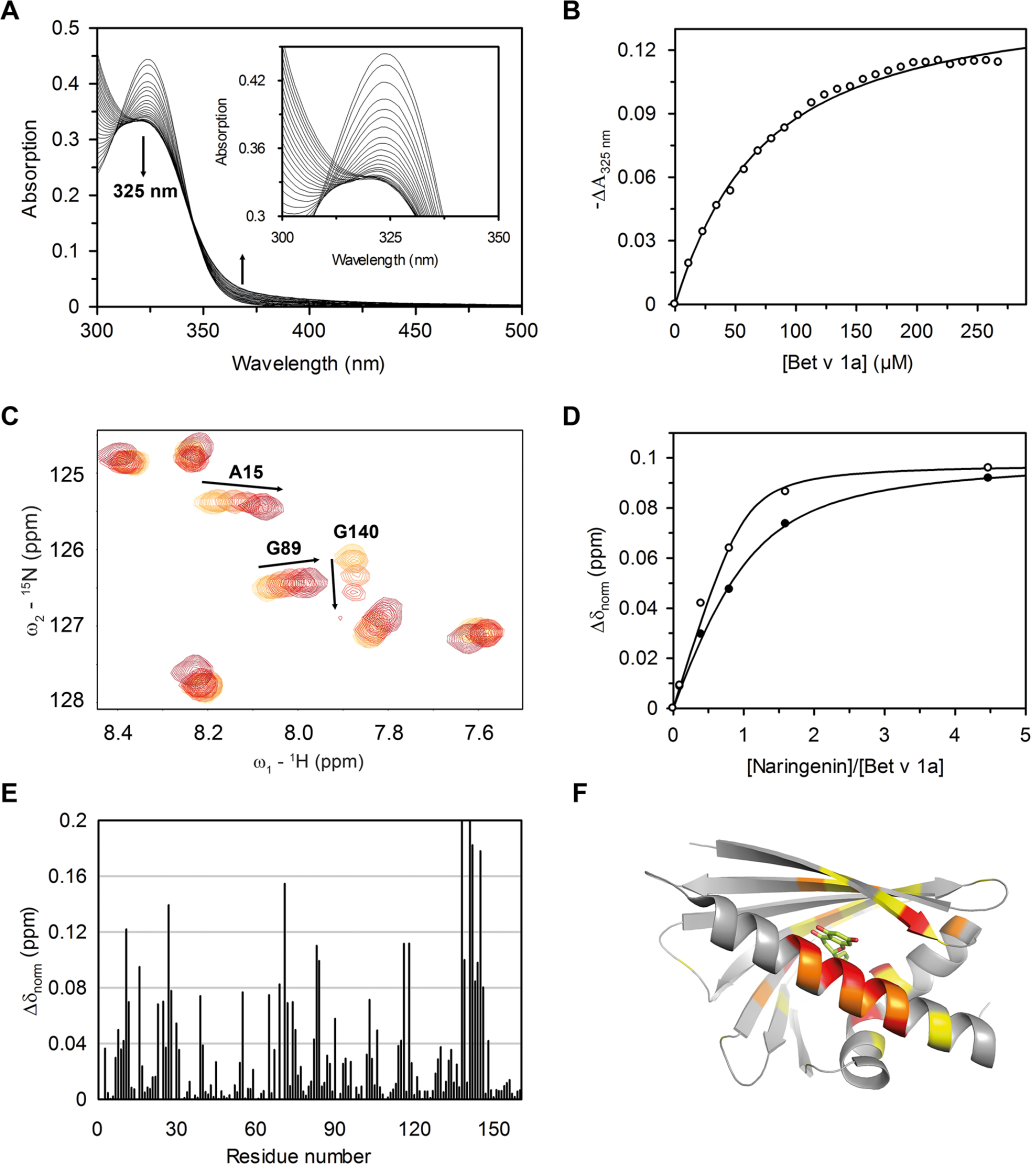
Binding of naringenin to Bet v 1a. A, UV/VIS spectra of the equilibrium titration of 20 *μ*M naringenin with Bet v 1a. All spectra were recorded at 298 K with 50 mM sodium phosphate, 50 mM NaCl at pH 7.0 and 10% DMSO as sample buffer. B, Absorbance changes at 325 nm plotted against the Bet v 1a concentration as shown for the data in A. The curve represents the best fit to Eq (1) resulting in a *K*_d_ value of 60.6 ± 3.2 *μ*M. C, Overlay of six ^1^H-^15^N HSQC spectra of 100 *μ*M Bet v 1a in the presence of increasing naringenin concentrations from light to dark red. The experiments were performed with a Bruker Avance 700 MHz spectrometer in 50 M sodium phosphate, 50 mM NaCl, pH 7.0 and 10% ^2^H_2_O at 298 K. Naringenin was added from a stock prepared in deuterated DMSO to a final excess of 1:4.5 over Bet v 1a and a final DMSO concentration of 10%. D, Chemical shift changes (Δδ_norm_) calculated with Eq (2) for residues A^15^ (○) and G^89^ (●) plotted against the ration of naringenin:Bet v 1a during titration. The curves represent the best fit to a quadric binding equation from the analysis software of NMRViewJ [89] (S1 Table). E, Calculated Δδ_norm_ values upon naringenin addition plotted against the Bet v 1a amino acid sequence and F, mapped on a cartoon representation of the complex structure of Bet v 1a:naringenin (pdb code: 4A87) with 0.04 ppm ≤ Δδ ≤ 0.08 ppm shown as yellow; 0.08 ppm ≤ Δδ ≤ 0.12 ppm shown as orange; and 0.12 ppm < Δδ shown as red. Bet v 1a in grey, naringenin in green sticks, oxygen in red.

A set of five different flavonoids was used to analyse the influence of number and position of hydroxyl groups of the flavonoid moiety during binding to Bet v 1 isoforms (Table 1 and S1A to S1E Fig). UV/VIS and chemical shift perturbation (CSP) measurements with ^1^H-^15^N HSQC NMR spectroscopy were performed to study affinities and binding sites of various flavonoids. The UV/VIS spectra from the titration experiment of naringenin and Bet v 1a show isosbestic points indicating a two-state binding process with a *K*_d_ of roughly 60 *μ*M (Fig 2A and 2B). In the ^1^H-^15^N HSQC spectra of ^15^N-Bet v 1a with increasing concentration of naringenin, the G^140^ resonance was in the intermediate exchange regime, but gradual CSPs were observed for the majority of affected resonances (Fig 2C, S1 Table), from which a *K*_d_ value of approximately 30 *μ*M could be estimated (Fig 2D and 2E). The CSP mapping on the Bet v 1a:naringenin structure (pdb code 4A87, [30]) agreed well with the results from X-ray crystallography (Fig 2F). We confirmed F^22^, Y^83^, I^102^, and E^141^ as interacting residues (S1 Table) with CSPs > 0.12 ppm and the reported change in side chain conformation of K^137^ [30] could also be observed as large CSP with a Δδ_norm_ value of 0.27 ppm.

**Table 1:** Dissociation constants for Bet v 1 isoform interaction with flavonoids and sugars.

		Dissociation constant *K*_d_ (*μ*M)		
Flavonoid	Method	Bet v 1a	Bet v 1m	Bet v 1d
Flavone	UV/VIS	n.a. ^**1**^	n.a.	- ^**2**^
	NMR	67.1±12.1	213.3±36.6	69.9±14.8
Naringenin	UV/VIS	60.6±3.2	28.1±0.8	37.7±6.4
	NMR	30.0±7.0	22.1±5.5	-
Fisetin	UV/VIS	14.3±1.1	68.6±12.4	13.9±2.1
	NMR	37.2±6.5	85.1±21.7	-
Quercetin	UV/VIS	9.2±0.6	26.5±1.5	10.2±1.0
	NMR	31.4±10.3	65.8±8.2	-
Myricetin	UV/VIS	4.2±0.7	n.a.	1.2±0.2
	NMR	14.6±6.5	99.3±19.4	-
Glucose	UV/VIS	-	-	-
	NMR	No binding	No binding	No binding
Galactose	UV/VIS	-	-	-
	NMR	No binding	No binding	No binding
Q3OGlc	UV/VIS	n.a.	n.a.	-
	NMR	288.4±24.0	<5	No binding
	Docking	-	0.2–6.1	-
Q3OGal	UV/VIS	n.a.	n.a.	-
	NMR	<5	<5	No binding
	Docking	3.2–14.8	0.4–10.4	-
Q3OS	UV/VIS	n.a.	n.a.	-
	NMR	<1	No binding	No binding
	Fluorescence	0.57 [17]	-	-
	Docking	0.1–1.7	-	-

K_d_ values from UV/VIS titration experiments were determined by non-linear regression analysis. The error represents the standard error of the best fit according to Eq 1. The dissociation constants determined with NMR spectroscopy represent an averaged K_d app_ value of all analysable residues showing CSPs > 0.08 ppm (S1 to S3 Tables) with the corresponding standard deviation. K_d_ ranges from docking simulation were obtained from binding energies for ligands docked inside the hydrophobic pocket of Bet v 1a and Bet v 1m.

^1^ not analysable (n.a.)

^2^ not measured (-)

The *K*_d_ values of all tested flavonoids were in the medium to low micromolar range (Table 1, S1 to S3 Tables). We observed shifts of the UV/VIS absorption maxima and isosbestic points in the spectra upon Bet v 1 addition for all isoforms and flavonoids (S4 Table). During ^1^H-^15^N HSQC titration, the majority of affected Bet v 1 resonances were in the fast exchange regime with the highest *K*_d_ generally for the non-hydroxylated flavone. Thereby, the significant CSPs obtained during titration were generally spread over the sequence of each isoform, making it difficult to predict a precise binding site for flavone. Due to hydrophobic interactions, flavone seems to bind more flexibly and somewhat more weakly inside the hydrophobic pocket.

In general, we obtained the best results (lowest standard error) for our titration experiments by fitting the data to an equation corresponding to a simple bimolecular reaction (Eq 1 and a similar equation provided by the NMRviewJ software). Prior experiments on flavonoid binding to other allergens of the PR-10 class performed so far also suggested a single site binding scheme to be valid [8,30]. Therefore, it seems as there is only one binding site for flavonoids inside the Bet v 1 hydrophobic pocket.

While the position of hydroxyl groups is insignificant, the addition of such leads to a significant decrease of *K*_d_ for flavonoids interacting with Bet v 1 isoforms a and d. Myricetin contains six hydroxyl groups and shows a 15-fold higher affinity to Bet v 1a (4.2 *μ*M) and an even 60-fold higher affinity to Bet v 1d (1.2 *μ*M) than flavone (Table 1). Those affinities are characteristic for a change of resonance positions and shapes in the form in the fast exchange regime to the intermediate exchange regime on the NMR time scale ([38], S1 Table). Accordingly, in the presence of myricetin, almost half of the affected resonances (11 of 28 residues) of Bet v 1a are in the intermediate exchange regime (S1 Table). Bet v 1m generally shows lower affinities towards the tested flavonoids compared to Bet v 1 isoforms a and d. Furthermore, the *K*_d_ values seem to be independent of the number of flavonoid hydroxyl groups. However, the presence of a hydroxyl group at C5’ in the B-ring of fisetin and myricetin decreases the affinity towards Bet v 1m compared to naringenin and quercetin (Table 1).

The interaction surfaces of all flavonoids are located inside the hydrophobic pocket of Bet v 1 but vary between Bet v 1a (T^7^ to S^11^, I^23^ to N^28^, F^64^, G^89^ to I^91^, I^102^, K^115^ to N^118^, K^137^ to E^141^, and R^145^) and Bet v 1m (T^57^, G^89^ to G^92^, I^102^, K^137^ to L^143^). Most likely, flavonoids enter the hydrophobic pocket *via* one of the two gaps formed by the mostly nonpolar residues F^62^, P^63^, F^64^, P^90^, Q^132^, A^135^, S^136^, and M^139^ (entrance 1) or by residues I^23^, L^24^, D^25^, D^27^, T^52^, K^54^, Y^81^, and I^102^. The third gap, Y^5^, T^7^, V^133^, and K^137^ with a diameter of ~6 Å, is probably too small for flavonoids to enter the cavity [30].

Despite the observed differences between the three isoforms with respect to binding of unglycosylated flavonoids, the hydrophobic cavity of Bet v 1 isoforms seem to be promiscuous acceptors of small hydrophobic and amphiphilic molecules *in vitro*. However, the vast majority of naturally occurring flavonoids are modified with additional functional groups such as methyl ether groups, glycosylations, or combinations of these [39]. In addition, the low water solubility of unglycosylated flavonoids [40] and their low potential physiological concentration in pollen [41] is not necessarily indicative of a major physiological importance of these complexes.

### Binding of glycosylated flavonoids is governed by the sugar moiety

As no isoform-specific binding pattern for unglycosylated flavonoids could be derived, we focused on the sugar moiety of the quercetin glycosides quercetin-3-O-sophoroside (Q3OS), quercetin-3-O-glucoside (Q3OGlc), and quercetin-3-O-galactoside (Q3OGal) as binding partners of Bet v 1 isoforms (Table 1 and S1F to S1H Fig). UV/VIS absorption spectra show maxima of different intensities at physiological pH for Q3OGlc at 364 nm, and for Q3OGal and Q3OS at 358 nm (Fig 1B), but the spectral changes on Bet v 1 binding were too small to be analysed with confidence. Thus we resorted to ^1^H-^15^N HSQC spectroscopy for further studies.

**Fig 3:**
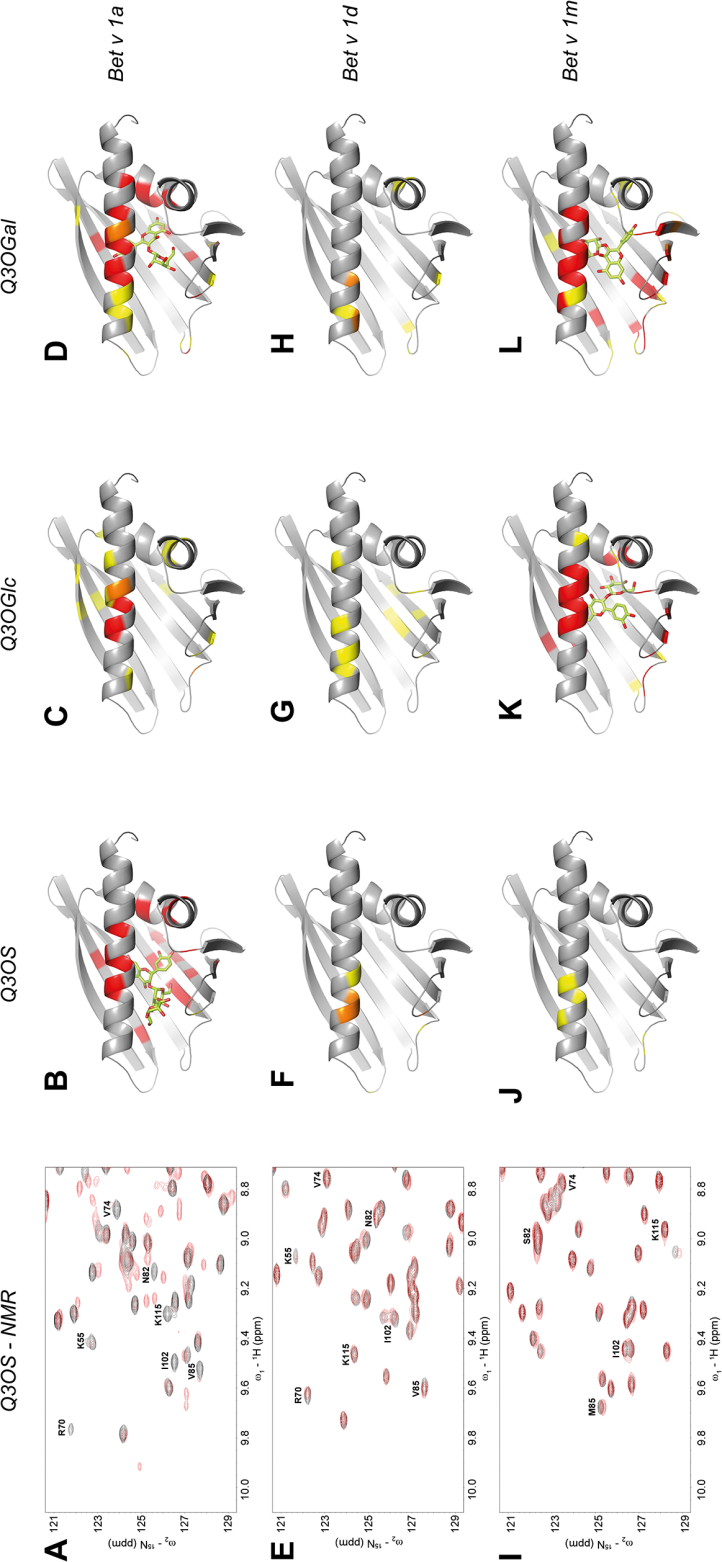
Binding of quercetin glycosides to Bet v 1 isoforms. All experiments were performed with 50 *μ*M (Q3OS) or 100 *μ*M (Q3OGlc, Q3OGal) ^15^N-uniformly labelled Bet v 1 isoforms at 298 K in 50 mM sodium phosphate buffer, 50 mM NaCl at pH 7.0, and 10% ^2^H_2_O with Bruker Avance 700 MHz and Avance 800 MHz spectrometers. Chemical shift changes were mapped on Bet v 1a (pdb code: 1BV1, grey) or models of Bet v 1d and Bet v 1m as in Fig 2F. Models of Bet v 1d and Bet v 1m were created using the Phyre server [92]. Docked ligands [93] are illustrated in green sticks, oxygen in red. A Overlay of two ^1^H-^15^N HSQC spectra of Bet v 1a in the absence (black) and presence of a 15-fold excess of Q3OS (red). B Disappearing resonances after addition of Q3OS mapped on Bet v 1a in red. Q3OS is docked inside the hydrophobic pocket [17]. C Mapping of chemical shift changes of (weak) Q3OClc or D (strong) Q3OGal interaction on Bet v 1a. E Overlay of two ^1^H-^15^N HSQC spectra of Bet v 1d in the absence (black) and presence of a 15-fold excess of Q3OS (red) and F occurring chemical shift changes mapped on a model of Bet v 1d. Weak affinity is observed for interaction of Bet v 1d with G Q3OGlc of H Q3OGal. I Overlay of two ^1^H-^15^N HSQC spectra of Bet v 1m in the absence (black) and presence of a 15-fold excess of Q3OS (red) and J occurring chemical shift changes mapped on a model of Bet v 1m. High affinity is observed for the interaction of Bet v 1m with K Q3OGlc and L Q3OGal. Regions of the ^1^H-^15^N HSQC spectra during titration of Bet v 1d or Bet v 1m with Q3OGlc and Q3OGal are provided in the S1 Fig

Titration of Bet v 1a with Q3OS resulted in a change of resonance positions on the intermediate to slow exchange limit on the NMR time-scale for 16 residues (F^22^, L^29^, I^38^, K^55^, R^70^, E^73^, V^74^, N^82^, S^84^, V^85^, K^115^, Y^120^, K^137^, E^138^, G^140^, and L^144^), with a resulting *K*_d_ of 566 ± 85 nM ([17], Fig 3A and 3B, S1 Table). Although Q3OGlc is simply shortened by a single glucose moiety compared to Q3OS, the Bet v 1a:Q3OGlc *K*_d_ of 288 *μ*M is three orders of magnitude higher than that of Bet v 1a:Q3OS (Fig 3C and S2A Fig). In contrast, Bet v 1a shows high affinity to Q3OGal with resonances of 16 residues in intermediate exchange (F^22^, I^23^, G^26^, K^54^, F^64^, R^70^, E^73^, D^93^, K^115^, S^136^ to E^141^, and L^144^) and 11 residues in the fast exchange regime (T^10^, I^53^, T^66^, G^92^, L^95^, V^128^, Q^132^, V^133^, A^135^, T^142^, and V^147^) showing CSPs > 0.04 ppm (Fig 3D and S2B Fig, S1 Table). According to docking simulations, Q3OGal binds in the hydrophobic pocket of Bet v 1a, with the sugar moiety either completely inside or at the opening of the pocket (entry ε1, [30]) at the flexible loop connecting β7 with α3. Since we observed the majority of affected resonances in the intermediate exchange regime, we concluded that the affinity of Bet v 1a to Q3OGal is higher than for its aglycon quercetin (9.6 *μ*M) and estimated the *K*_d_-value < 5 *μ*M. Affinity scores of the models resulted in *K*_d_ values from 3.2 *μ*M to 14.8 *μ*M (Table 1). Obviously, stereochemical changes in the sugar moiety of flavonol glycosides can strongly influence the affinity to Bet v 1a.

Although Bet v 1d binds flavonoids with affinities comparable to those of Bet v 1a (Table 1), it shows only very weak affinity for the glycosylated flavonoids that we have analysed here. Remarkably, even a 15-fold excess of Q3OS, Q3OGlc or Q3OGal (Fig 3E to 3H, S2C and S2D Fig) did not produce significant CSPs for Bet v 1d.

Furthermore, titration of Bet v 1m with Q3OS also did not lead to significant CSPs (Fig 3I and 3J), suggesting that Bet v 1a:Q3OS formation is highly specific. However, in contrast to Bet v 1a and d, Bet v 1m strongly binds to Q3OGlc (estimated *K*_d_ < 5 *μ*M), with resonances of 21 residues in intermediate exchange (E^6^, I^23^, G^26^, I^38^, T^57^, F^64^, Y^66^, G^89^ to G^92^, I^98^, and I^136^ to L^144^) and four residues (A^34^, E^87^, E^96^, and V^147^) with CSPs > 0.04 ppm (Fig 3K and S2E Fig, S2 Table). The docking simulation suggested Q3OGlc to bind in the hydrophobic pocket of Bet v 1m with *K*_d_ values of 0.4 *μ*M to 10.4 *μ*M. Bet v 1m also shows high affinity for Q3OGal with 21 intermediate exchanging residues (I^38^, S^39^, T^57^, F^64^, Y^66^, M^85^ to E^87^, G^89^ to G^92^, E^96^, K^134^, I^136^ to E^141^, L^143^, and L^144^) and 14 residues (D^25^, A^34^, A^37^, V^41^, N^47^, I^56^, E^87^, G^88^, T^94^, L^95^, K^115^, T^122^, K^123^, and A^135^) with CSPs > 0.04 ppm (Fig 3L and S2F Fig, S2 Table) and *K*_d_ values obtained from docking simulations between 0.2 *μ*M and 6.1 *μ*M (Table 1).

Although glycosylation drastically changed the binding behaviour of quercetin to the various Bet v 1 isoforms, glucose and galactose alone showed no detectable affinity to any isoform (Table 1).

Bet v 1d varies in seven amino acids (T7I, F30V, S57N, I91V, S112C, I113V, and D125N; Fig 1C) compared to Bet v 1a. Thus, strong specific binding and virtual lack of such is achieved by variation of just seven or even fewer amino acids. None of those seven variable residues, however, is directly involved in Q3OS or Q3OGal binding in Bet v 1a or is part of the amino acids which form the potential entrances. T^7^ is part of the third opening in Bet v 1a, which is presumably too small for glycosylated flavonoids entrance. The loss of affinity might be explained by a slightly different structural arrangement of Bet v 1d, which could result in variations in the openings to the hydrophobic pocket. In contrast to Bet v 1d, Bet v 1m shows four variations in entrance 1 (F62S, P90A, Q132H, and S136I compared to Bet v 1a; Fig 1C) which are likely to directly block the access route for Q3OS, but not for Q3OGlc and Q3OGal, into the hydrophobic pocket. Substitutions of amino acids in the C-terminal helix (S136I, M139K, and T142A) could contribute to an increased affinity to Q3OGlc as compared to Bet v 1a as the C-terminal helix determines size and character of the hydrophobic cavity in PR-10 proteins [33].

In addition to structural aspects, a phenomenon known as enthalpy-entropy compensation [42] can explain the binding behaviour of the isoforms to glycosylated flavonoids and the sugars alone. Upon Bet v 1 isoform–ligand complexation, water molecules that form the hydration shell of the sugar moiety and the binding cavity will tend to escape to the bulk with a concomitant decrease or increase in entropic energy contribution, depending on the pre-existing molecular interactions. This event is accompanied by the increase or decrease of degrees of freedom for the ligand and the residues forming the binding site. The setup of solvent clusters on the surface of the protein-ligand complex also contributes to the overall binding affinity with enthalpy/entropy gains (Bet v 1a:Q3OS or Q3OGal; Bet v 1m:Q3OGlc or Q3OGal), penalties (Bet v 1a:Q3OGlc), or even complete abolishment of observable binding (Bet v 1d:Q3OS, Q3OGlc or Q3OGal; Bet v 1m:Q3OS) compared to the aglycon quercetin. Similar effects have been reported and seem to be generally characteristic for each ligand/receptor involved [43–45]. In addition, glucose and galactose alone showed no detectable affinity to any isoform (Table 1). The potential enthalpy gains upon carbohydrate interaction with proteins are often counteracted by the above described change of entropy [42], resulting in the abolishment of binding. We observed this effect already for the binding of sophorose to Bet v 1a [17].

In summary, our results firmly suggest that Bet v 1:ligand binding is isoform-specific and that the binding specificity is entropically driven by the sugar moiety. Glycosylation of quercetin can thereby significantly increase the affinity compared to the aglycon (Table 1). The hydrophobic pockets formed by Bet v 1 isoforms are obviously designed for specific discrimination between the sugar moieties of glycosylated flavonoids.

### Allergenicity of Bet v 1 isoforms is unaffected by ligands

**Fig 4:**
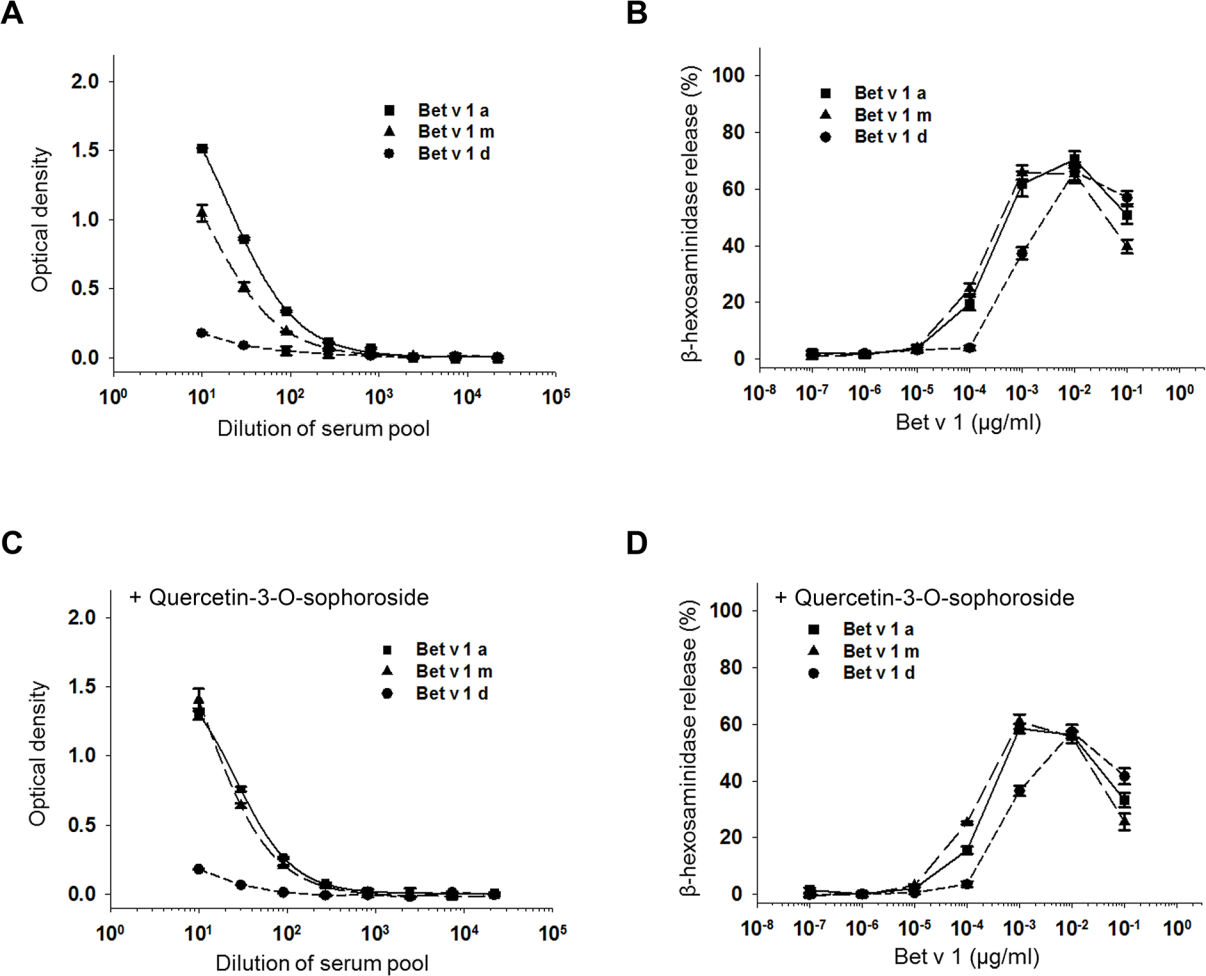
Interaction of Bet v 1 isoforms with serum IgE in the absence and presence of Q3OS. A, Binding of serial dilutions of pool serum IgE to equimolar amounts of surface-coated Bet v 1a, Bet v 1d, and Bet v 1m. Allergen-specific human IgE was detected with a horseradish peroxidase-conjugated mouse anti-human IgE antibody. As substrate 3,3′,5,5′-tetramethylbenzidine was used and the absorbance at 450 nm was measured after stopping the reaction with 25% H_2_SO_4_. B, Mediator release induced by recombinant Bet v 1 isoforms. Humanized rat basophil leukemia cells were sensitized with a pool of human birch-specific sera. Cross-linking of membrane-bound human IgE by IgE-Bet v 1 isoform interaction and subsequent release of β-hexosaminidase was determined with serial dilutions of Bet v 1 a, d and m. The β-hexosaminidase activity in the culture supernatants was quantified by photometric measurements. The percentage of β-hexosaminidase activity relative to cells lysed with Triton X-100 was calculated and corrected for spontaneous release. C, Binding of serial dilutions of pool serum IgE to equimolar amounts of surface-coated Bet v 1a, Bet v 1d, and Bet v 1m (as described in A) and D, mediator release (as described in B) in the presence of a 5-molar excess of Q3OS.

Bet v 1 isoforms can be grouped into three classes with molecules showing high (isoforms a, e, and j), intermediate (isoforms b, c, and f), and low/no IgE-binding activities (d, g, and l) [35]. A study on the modulation of IgE reactivity by site-directed mutagenesis revealed a limited number of crucial amino acid positions (residues F^30^, S^57^, S^112^, I^113^, and D^125^ in the Bet v 1a sequence) that strongly influence IgE binding [36]. Although Bet v 1 isoforms d, g, and l are highly similar in sequence to Bet v 1a (95.6%, 95.0%, 94.3% identity, respectively), those hypoallergenic isoforms show variations in each of these positions. A small subset of critical amino acids can drastically modulate the binding of IgE to an epitope and consequently change the allergenicity of Bet v 1 isoforms as exemplified by Bet v 1 isoforms a and d [35,46]. In the absence of ligands, we observed comparable IgE interactions (Fig 4A) and mediator release activities (Fig 4B) for isoforms a and m as measured by indirect ELISA and β-hexosaminidase release from humanized rat basophil leukaemia (RBL) cells. Sequence and allergenicity of Bet v 1m and the intermediate IgE-binding isoform Bet v 1b are nearly identical (Bet v 1.0201, 98.1% identity; [23]). The IgE-binding capacity of Bet v 1d is only marginal in the ELISA, and consequently an approximately 10-fold shift to a higher Bet v 1d concentration is needed for half-maximum release of β-hexosaminidase in comparison to the other isoforms (Fig 4A and 4B). Comparable results concerning the allergenicity of these Bet v 1 isoforms were also obtained in previous experiment [35,36,46].

X-ray crystallography revealed that Bet v 1:ligand interaction could lead to an increase in volume of the hydrophobic pocket, thus altering the protein surface [30,37], an effect that was hypothesized to influence IgE epitopes. Our results, however, do not indicate any significant influence of high-affinity ligands on the IgE binding properties of Bet v 1. Presence of a 5-fold molar excess of Q3OS does not significantly influence the interaction of IgE with any of the three isoforms (Fig 4C and 4D), and rutin, quercetin, Q3OGlc, Q3OGal, and sophorose did not modify IgE-binding of the Bet v 1 isoforms either (S3 Fig). Our results are in agreement with a recent study on the influence of deoxycholate on the allergenic properties of Bet v 1a [47].

Although recognition of an allergen by IgE is the key step in the allergic response, numerous other factors such as functional activity, presence of infective agents or chemical substances can induce non-specific inflammatory responses or will augment the immunological shift towards an allergic reaction [48]. We suggest the lack of a direct ligand effect on IgE recognition of Bet v 1, but leave open the possibility of indirect influences or sensitization [49]. Indeed, flavonoids influence the inflammatory pathway in human cells [50], and their uptake by the human body may be facilitated by Bet v 1 [51,52].

### Bet v 1:flavonol-glycosides—adaptable sunscreens for birch pollen DNA?

The Bet v 1:Q3OS complex was suggested to protect pollen DNA from UV-damage, and the mixture of different isoforms was suggested to provide an individual fingerprint to prevent self-pollination [17]. Indeed, glycosylated flavonoids are common in plant pollen. Flavonol-3-O-glycosides, e. g., were found in pollen from alder, ragweed, buttercup, date palm, narrowleaf cattail, hazelnut, petunia, maize, and ophrys [11,53–60], and quercetin-3-O-glycosylgalactoside was identified in pollen from *Betula verrucosa* [12] along with the Bet v 1a ligand Q3OS. Interactions of glycosylated flavonoids with different Bet v 1 isoforms in combination with variations in the production and composition of isoforms during maturation of pollen are probably dependent on a set of parameters like climate, location, and solar radiation, as the Bet v 1 levels in pollen are not constant over time [61], show variable IgE reactivity [27], and vary geographically [26,62]. Upon UV-B radiation flavonoids (mostly quercetin derivatives) are produced to protect the DNA from radiation damage [63] and glycosylation increases the UV tolerance of a flavonoid compared to the corresponding aglycon [64,65]. As we observed a shift of the absorption maximum of quercetin depending on the sugar moiety (Fig 1B) and the absorption maxima of different unglycosylated flavonoids shift towards higher (myricetin, quercetin, fisetin) or lower (naringenin) wavelengths during UV/VIS titration with Bet v 1 isoforms (S4 Table), Bet v 1 complex formation combined with variation of isoform composition in pollen may be a means to expand or to optimize the absorption spectrum for sunlight-emitted UV-A radiation.

After maturation and before dispersing into the environment, the pollen dehydrate [66] to reduce their water content to 20% [67], thus forming highly viscous intracellular glass-like structures [68]. In this milieu of highly concentrated biomolecules, glycosylated flavonoids may be protected from degradation or chemical modulation by complex formation with Bet v 1.

Although flavonoids are considered most effective UV-B screening compounds because of their strong absorbance in the UV region [69], continuous UV-irradiation leads to their degradation [64]. Existence of functional complexes of glycosylated flavonoids and Bet v 1 in high concentration may serve as an important signal for unharmed pollen DNA as UV-damage of the flavonoid moiety may modify the complex and prevent pollination. The pollen–pistil interaction before fertilization comprises a series of complex cellular interactions involving a continuous exchange of signals between pollen and the pistil of the stigma [70,71]. Upon contact, birch pollen get rehydrated, and the Bet v 1-ligand complexes are released onto the stigma surface [10,66] with the specific mixture of the isoforms and ligands possibly serving as molecular fingerprints to prevent self-pollination.

This means that isoforms of PR-10-allergen do not simply just exist by chance, but have been selected through evolution with each isoform fulfilling a particular function. Isoforms from other Bet v 1 homologs like Ara h 8 [72,73], Dau c 1 [74,75], Api g 1 [76,77], Pru av 1 [78,79] or Fra a 1 [80,81] seem to have less diverse functions *in vivo* without the necessity to provide such a complex individual fingerprint. In those cases, the amount of (so far identified) genetically available and actually expressed isoforms seems to be significantly lower than observed for example for Mal d 1 in apple [82–87] or Bet v 1 in birch pollen [23,26,27].

## Materials and Methods

### Flavonoids

All nonglycosylated and monoglycosylated flavonoids as well as glucose and galactose were purchased in analytical grade from Sigma-Aldrich. Q3OS was obtained from ALNuMed (Germany) or AApin Chemicals Limited (UK).

### Protein preparation

The genes coding for Bet v 1d (Bet v 1.0102; UniProt P43177) and Bet v 1m (Bet v 1.0204; UniProt P43186) were purchased from GeneScript and cloned into the bacterial expression vector pET11a (Novagen) using the restriction enzymes NdeI and BamHI-HF (New England Biolabs). The expression for all isoforms was performed as previously described for Bet v 1a (Bet v 1.0101, UniProt P15494, [17]) with minor modifications. For purification, Bet v 1 isoforms d and m were regained from protein pellets after cell lysis with 50 mM sodium phosphate, pH 7.4, 200 mM NaCl, and 8 M urea and refolded by subsequently lowering the urea concentration during dialysis in 20 mM Hepes buffer, pH 8.0 and 500 mM NaCl at 4°C (Bet v 1d) or 20 mM Hepes buffer, pH 8.0 at RT (Bet v 1m).

Refolded Bet v 1d was further purified *via* hydrophobic interaction chromatography on a 4 ml octyl sepharose column (HiTrap, Octyl Fast flow, GE Healthcare) equilibrated with loading buffer (20 mM Hepes, pH 8.0, 1 M ammonium sulphate) and eluted stepwise with elution buffer (20 mM Hepes, pH 8.0). Refolded Bet v 1m was loaded on a 25 ml Q sepharose column (Q sepharose Fast flow, GE Healthcare) equilibrated with loading buffer (20 mM Hepes, pH 8.0) followed by elution with 20 mM Hepes, pH 8.0, 300 mM NaCl. Bet v 1a was purified as previously described [17]. Fractions containing the respective Bet v 1 isoform were pooled and dialyzed at 4°C against 50 mM sodium phosphate, pH 7.0, 50 mM NaCl, concentrated and stored at -80°C. Protein concentrations were determined by the DC protein assay (BioRad) and UV/VIS spectroscopy using the molar extinction coefficient ε_280_ = 10430 M^-1^ cm^-1^. Standard methods were used to analyse purity (SDS/PAGE), oligomeric state (size exclusion chromatography), and signal dispersion (^1^H-^15^N HSQC spectroscopy) of all isoforms (S4 Fig).

### UV/VIS spectroscopy

All flavonoids and Bet v 1 isoforms were dissolved in 50 mM sodium phosphate, 50 mM NaCl, 10% (v/v) DMSO, pH 7.0, to a final concentration of 10 to 20 *μ*M in 500 *μ*l. Absorption spectra from 200–800 nm were recorded at 25°C in a 1 cm quartz cuvette (Hellma GmbH) using a 8453 UV-visible spectrophotometer (Agilent).

To observe binding of Q3OS to Bet v 1 isoforms a, d, and m, 20 *μ*M Q3OS were initially incubated with 20 *μ*M of the respective isoform in buffer without DMSO for 30 min at room temperature (RT) in a total volume of 550 *μ*l. Samples were concentrated to a final volume of 100 *μ*l using a Vivaspin concentrator (Sartorius, molecular mass cut off 10 kDa). The concentrated samples where loaded on a G25 spin trap column (GE Healthcare) and eluted as described in the manual. Absorption spectra of the eluted fractions were normalized at 280 nm and set to zero at 700 nm.

To further characterize flavonoid binding to Bet v 1, titration experiments were performed by adding small amounts of concentrated Bet v 1 isoform a, d or m to different flavonoids. Changes of flavonoid absorption occurring at specific wavelengths were plotted against the protein concentration. Prior to curve-fitting, absorbance data were corrected for dilution. If possible, the equilibrium dissociation constant (*K*_d_) was determined by non-linear regression analysis of the data with GraFit-5 (Version 5.0, Erithacus Software, UK) using the following Eq (1):

$$\Delta \text{A}=\frac{{\Delta \text{A}}_{\text{max}}}{2\text{Q}}\left[\left({\text{B+Q+K}}_{\text{d}}\right)\text{-}\left({\left({\text{B+Q+K}}_{\text{d}}\right)}^{2}\text{-}{\left(4\text{BQ}\right)}^{0.5}\right)\right]$$ (1)

Δ*A*_max_, maximum change in absorbance at specific wavelengths; *B*, Bet v 1a concentration; *Q*, total flavonoid concentration.

## NMR spectroscopy

All NMR experiments were performed at 298 K in 50 mM sodium phosphate buffer, 50 mM NaCl, pH 7.0, 10% deuterium oxide (^2^H_2_O) with ^15^N-uniformly labelled Bet v 1 isoforms using Bruker Avance 700 MHz and Avance 800 MHz spectrometers with cryogenically cooled triple-resonance probes equipped with pulsed field-gradient capabilities. NMR data were processed using NMRPipe [88] and visualized with NMRViewJ [89]. Three-dimensional ^15^N-edited NOESY (nuclear Overhauser enhancement spectroscopy, mixing times 120 ms) experiments to assign chemical shifts were obtained with 500 *μ*M ^15^N-labeled samples of Bet v 1 isoform d or m and yielded 91% of assigned residues for Bet v 1d and 89% for Bet v 1m. The sequence-specific assignments of the amide resonances of Bet v 1a are reported elsewhere [90].

Interaction of Q3OS with the Bet v 1 isoforms was measured by incubating 700 *μ*M Q3OS with 50 *μ*M of each ^15^N-labeled Bet v 1 isoform in 50 mM sodium phosphate, 50 mM NaCl buffer, pH 7.0.

For titration experiments all other flavonoids were dissolved in deuterated DMSO, while glucose and galactose were dissolved in 50 mM sodium phosphate buffer, 50 mM NaCl, pH 7.0, 10% deuterium oxide and titrated stepwise to a final excess of up to 17-fold to protein samples (ca. 100 *μ*M). Final DMSO concentrations did not exceed 10% (v/v). Chemical shift perturbations caused by increasing DMSO concentrations during measurements were identified by titrating DMSO in comparable steps. CSPs for ligand binding were calculated based on Eq (2):

$${\Delta \delta }_{\text{norm}}=\sqrt{{\left({\Delta \delta }_{\text{HN}}\right)}^{2}+{\left(0.1\cdot {\Delta \delta }_{\text{N}}\right)}^{2}}$$ (2)

Δδ_HN_ and Δδ_N_, chemical shift differences of amide proton and nitrogen resonances, respectively, in ppm.

*K*_d_ values for flavonoid binding were determined with NMRViewJ [89]. All analysable amino acid residues that were unaffected by DMSO addition and showing CSPs > 0.08 ppm were fitted to a quadratic binding curve with default settings, and an average *K*_d app_ was calculated (Table 1 and S1–S3 Tables). The CSPs of all residues showing CSPs > 0.04 ppm were mapped either on models of Bet v 1d and Bet v 1m or on the Bet v 1a structure (pdb code 1BV1, [29]).

## Sequence alignments, modelling and docking simulation

Sequence alignments of the Bet v 1 isoforms a, d and m were performed with ClustalW [91]. Models of Bet v 1d and Bet v 1m were created using the Phyre2 server [92]. The calculated models are based on the structural fold of PR-10 proteins with a confidence of 99% and a coverage of 92% (Bet v 1d) and 87% (Bet v 1m) compared to the template sequence. We used AutoDockVina [93] to dock ligands into the hydrophobic pocket of Bet v 1a and the model of Bet v 1m. The PDB files for Q3OGlc and Q3OGal were created with ProDrg [94]. Furthermore, input files for Bet v 1a (pdb code 1BV1), the model of Bet v 1m, Q3OGlc, and Q3OGal were generated with AutoDockTools [95]. The grid box (2.0 nm×2.4 nm×2.8 nm, or 13.44 nm^3^) was centred over the hydrophobic pocket of the isoforms and AutoDockVina was run with default settings. Affinity scores were given by AutoDockVina as binding energies (*Δ*G), which were subsequently used to calculate an equilibrium dissociation constant by Eq (3) with R = 0.001968 kcal·mol^−1^·K^−1^ and T = 298.15 K:

$${\text{K}}_{\text{d}}={\text{e}}^{\frac{\text{-}\Delta \text{G}}{\text{RT}}}$$ (3)

Ligand docking was performed only if more than five amino acids with Δδ ≥ 0.12 ppm or intermediate exchange rates were observed during NMR titrations. The output of the docking simulation lists up to nine energetically most favourable orientations of the respective ligand in the Bet v 1 pocket. The models in best agreement with our experimental NMR data were chosen to illustrate ligand binding to the Bet v 1 isoforms a or m.

### Sera used in the study

Fifteen sera of birch pollen-allergic subjects were collected, tested, and pooled according to the guideline of the European Medicines Agency (EMEA/CHMP/BWP/304831/2007). The serum pool is routinely used for batch-release testing of birch pollen-derived allergenic products at the Paul-Ehrlich-Institut. The same serum pool was used for both, ELISA and mediator release assays.

### Indirect ELISA for IgE binding to Bet v 1 isoforms

For IgE-ELISA experiments, Maxisorp plates (Nunc *via* Fisher Scientific) were coated overnight at room temperature with 50 ng/100 *μ*l recombinant Bet v 1 isoforms a, d, or m with a 5-fold molar excess of quercetin-3-O-sophorose, rutin, quercetin, quercetin-3-O-glucoside, quercetin-3-O-galactoside, or sophorose, respectively, in phosphate-buffered saline (PBS). After blocking with PBS containing 2% bovine serum albumin (BSA) these plates and an uncoated control were incubated with a dilution series of a serum pool of birch-pollen allergic subjects for 1 h at room temperature with PBS containing 0.05% Tween 20 and 0.1% BSA. Allergen-specific human IgE was detected with a horseradish peroxidase-conjugated mouse anti-human IgE antibody (Clone B3102E8, Southern biotech via Biozol, Eching, Germany) diluted 1:1000. 3,3′,5,5′-tetramethylbenzidine (Roth, Karlsruhe) was used as substrate for horseradish peroxidase, and the absorbance at 450 nm was measured after stopping the reaction with 25% H_2_SO_4_.

### β-Hexosaminidase release from humanized rat basophil leukemia (RBL) cells

The mediator release assay was performed according to an established protocol [96]. Briefly, RBL cells expressing the α-chain of the high-affinity receptor for human IgE were sensitized overnight at 37°C (5%CO_2_) with a serum pool of birch pollen-allergic subjects (diluted 1:40 in culture medium). After washing, cells were stimulated with serial dilutions of Bet v 1 isoforms a, d, or m in Tyrode's buffer containing 50% ^2^H_2_O. For complex formation, the Bet v 1 isoforms were incubated overnight with a 5-fold molar excess of Q3OS, rutin, quercetin, Q3OGlc, Q3OGal, or sophorose, respectively, before stimulating the cells. Degranulation was quantified by photometric measurement of β-hexosaminidase activity in the culture supernatants. The percentage of β-hexosaminidase activity relative to cells lysed with Triton X-100 (Sigma-Aldrich, Steinheim, Germany) was calculated and corrected for spontaneous release (sensitized cells without allergen).

## Supporting Information

S1 Fig Chemical structures of flavonoids used in this study. **A** flavone, **B** naringenin, **C** fisetin, **D** quercetin, **E** myricetin, **F** quercetin-3-O-glucoside, **G** quercetin-3-O-galactoside, **H** quercetin-3-O-sophoroside.
(TIF)

S2 Fig NMR titration experiments of Q3OGlc and Q3OGal with the Bet v 1 isoforms. The experiments were performed with 100 *μ*M ^15^N-uniformly labelled Bet v 1 isoforms at 298 K in 50 mM sodium phosphate buffer, 50 mM NaCl at pH 7.0, and 10% ^2^H_2_O with Bruker Avance 700 MHz and Avance 800 MHz spectrometers. Q3OGlc and Q3OGal were dissolved in deuterated DMSO and titrated stepwise to a final excess of up to 1:17 to protein samples. Final DMSO concentrations did not exceed 10% (v/v). Spectra are illustrated in a divergent colour scheme from red (absence of ligand) to blue (final excess of ligand). Intermediate exchanging residues are labelled. Titration experiments of Bet v 1a with **A** Q3OGlc and **B** Q3OGal, Bet v 1d with **C** Q3OGlc, **D** Q3OGal and Bet v1m with **E** Q3OGlc and **F** Q3OGal.
(TIF)

S3 Fig Interaction of Bet v 1 isoforms with IgE in the presence of different flavonoids and sophorose. The left panel shows binding of serial dilutions of serum IgE to equimolar amounts of surface-coated Bet v 1a (■), Bet v 1d (●), and Bet v 1m (▲) with 5-molar excess of **A** quercetin, **C** Q3OGlc, **E** Q3OGal, **G** sophorose, and **I** rutin respectively. Mediator release induced by recombinant Bet v 1 isoforms is illustrated in the right panel. Humanized RBL cells were sensitized with a pool of human birch-specific sera. Cross-linking of membrane-bound human IgE by IgE-Bet v 1 isoform interaction and subsequent release of β-hexosaminidase was determined with serial dilutions of Bet v 1a (■), Bet v 1d (●), and Bet v 1m (▲) with 5-molar excess of **B** quercetin, **D** Q3OGlc, **F** Q3OGal, **H** sophorose, and **J** rutin respectively.
(TIF)

S4 Fig Protein analytics. **A** SDS/PAGE on 19% gels of ca. 1 *μ*g Bet v 1 isoforms (MW 17.4 kDa) after purification. M, molecular-mass standard (Low Range, Bio-Rad Laboratories). **B** SEC of the isoforms performed with a Superdex S75 GL 10/300 column (total bed volume: 24 ml; GE Healthcare) in 50 mM sodium phosphate, 50 mM NaCl, pH 7.0 at RT. Column calibration was performed with conalbumin (75.0 kDa), ovalbumin (43.0 kDa), carbonic anhydrase (29.0 kDa) and ribonuclease (13.7 kDa). The elution profile of 0.5 mg Bet v 1a is shown in black, 0.25 mg of Bet v 1d in red and 2.4 mg of Bet v 1m in blue. The peaks correspond to monomeric proteins with molecular masses of 19.66 kDa (Bet v 1a), 17.63 kDa (Bet v 1d) and 19.74 kDa (Bet v 1m). **C** ^1^H-^15^N HSQC spectra of 100 *μ*M Bet v 1a (black), Bet v 1d (red) and Bet v 1m (blue) in 50 mM sodium phosphate, 50 mM NaCl, pH 7.0 and 10% ^2^H_2_O at 298 K.
(TIF)

S1 Table Bet v 1a residues affected from addition of flavonoids with CSPs showing Δδ_norm_ > 0.08 ppm. (DOCX)

S2 Table Bet v 1m residues affected from addition of flavonoids with CSPs showing Δδ_norm_ > 0.08 ppm. (DOCX)

S3 Table Bet v 1d residues affected from addition of flavonoids with CSPs showing Δδ_norm_ > 0.08 ppm. (DOCX)

S4 Table Absorption maxima of unglycosylated flavonoids and their Bet v 1-complexes. (DOCX)
