# Supplementary figures and images for: Ligand Recognition of the Major Birch Pollen Allergen Bet v 1 is Isoform Dependent
Source: PLoS One. 2015 Jun 4;10(6):e0128677. doi: 10.1371/journal.pone.0128677 (PMC4456386; doi:10.1371/journal.pone.0128677)

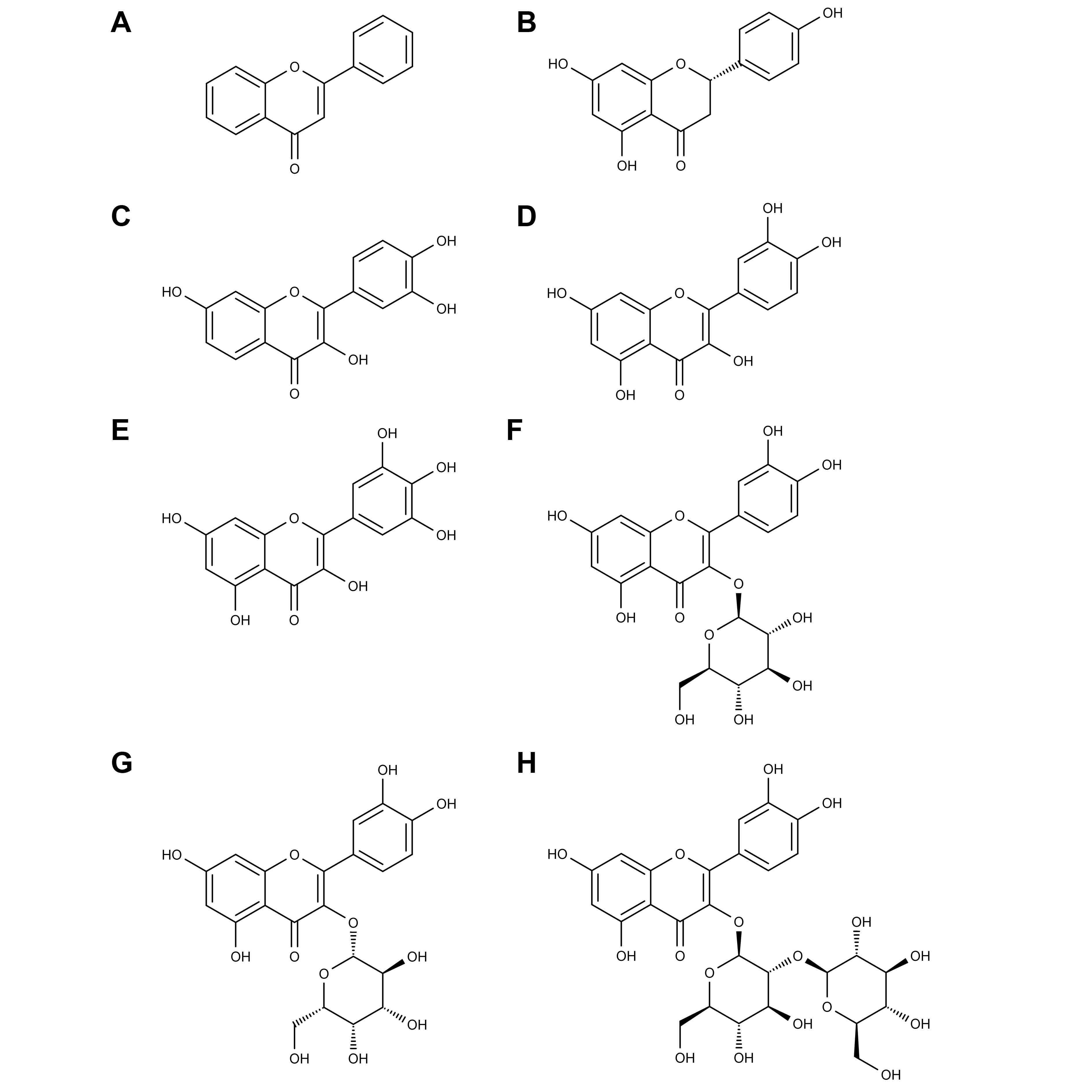

Supplement: S1 Fig — A flavone, B naringenin, C fisetin, D quercetin, E myricetin, F quercetin-3-O-glucoside, G quercetin-3-O-galactoside, H quercetin-3-O-sophoroside. (TIF) [file pone.0128677.s001.tif]

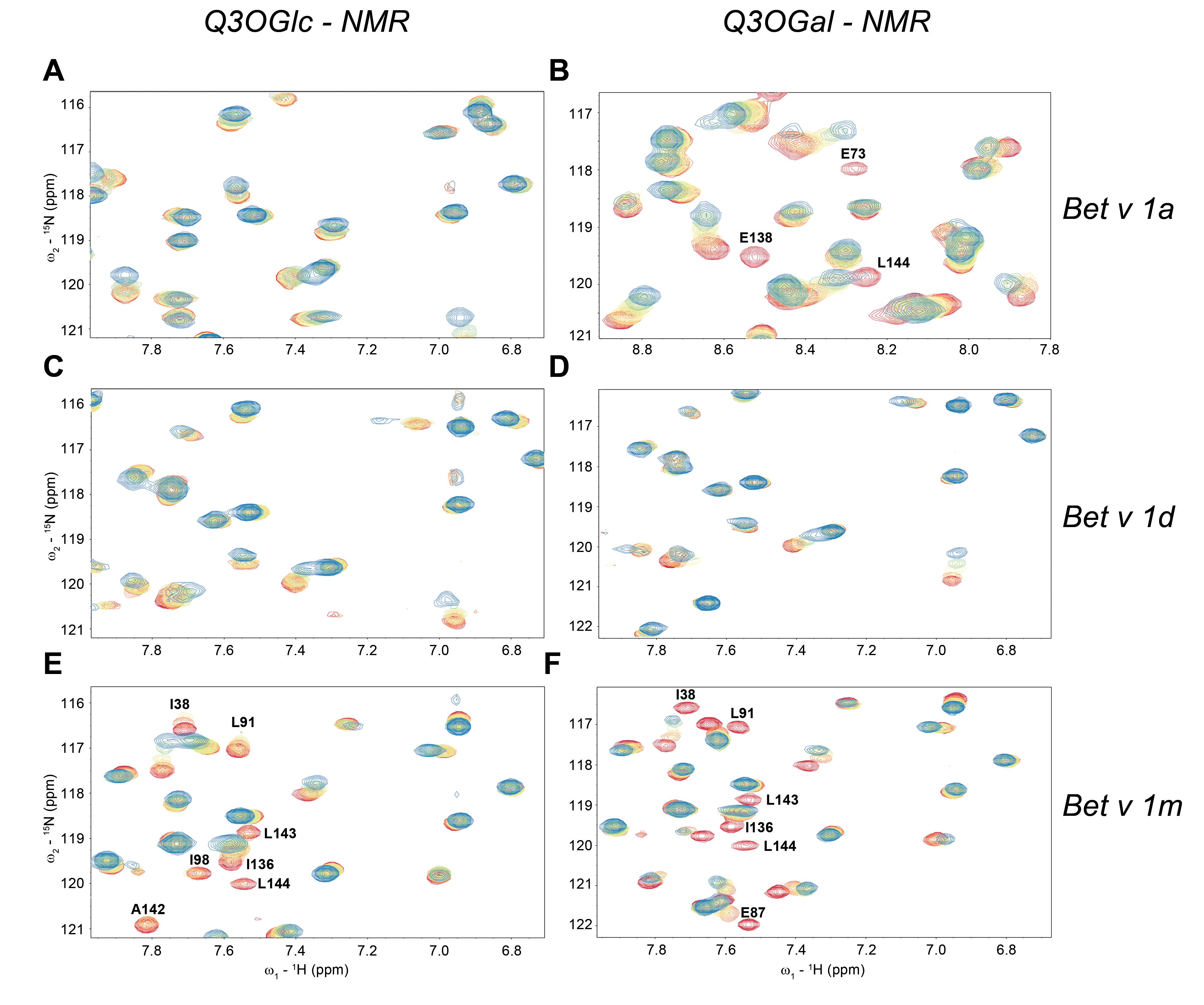

Supplement: S2 Fig — The experiments were performed with 100 μM 15N-uniformly labelled Bet v 1 isoforms at 298 K in 50 mM sodium phosphate buffer, 50 mM NaCl at pH 7.0, and 10% 2H2O with Bruker Avance 700 MHz and Avance 800 MHz spectrometers. Q3OGlc and Q3OGal were dissolved in deuterated DMSO and titrated stepwise to a final excess of up to 1:17 to protein samples. Final DMSO concentrations did not exceed 10% (v/v). Spectra are illustrated in a divergent colour scheme from red (absence of ligand) to blue (final excess of ligand). Intermediate exchanging residues are labelled. Titration experiments of Bet v 1a with A Q3OGlc and B Q3OGal, Bet v 1d with C Q3OGlc, D Q3OGal and Bet v1m with E Q3OGlc and F Q3OGal. (TIF) [file pone.0128677.s002.tif]

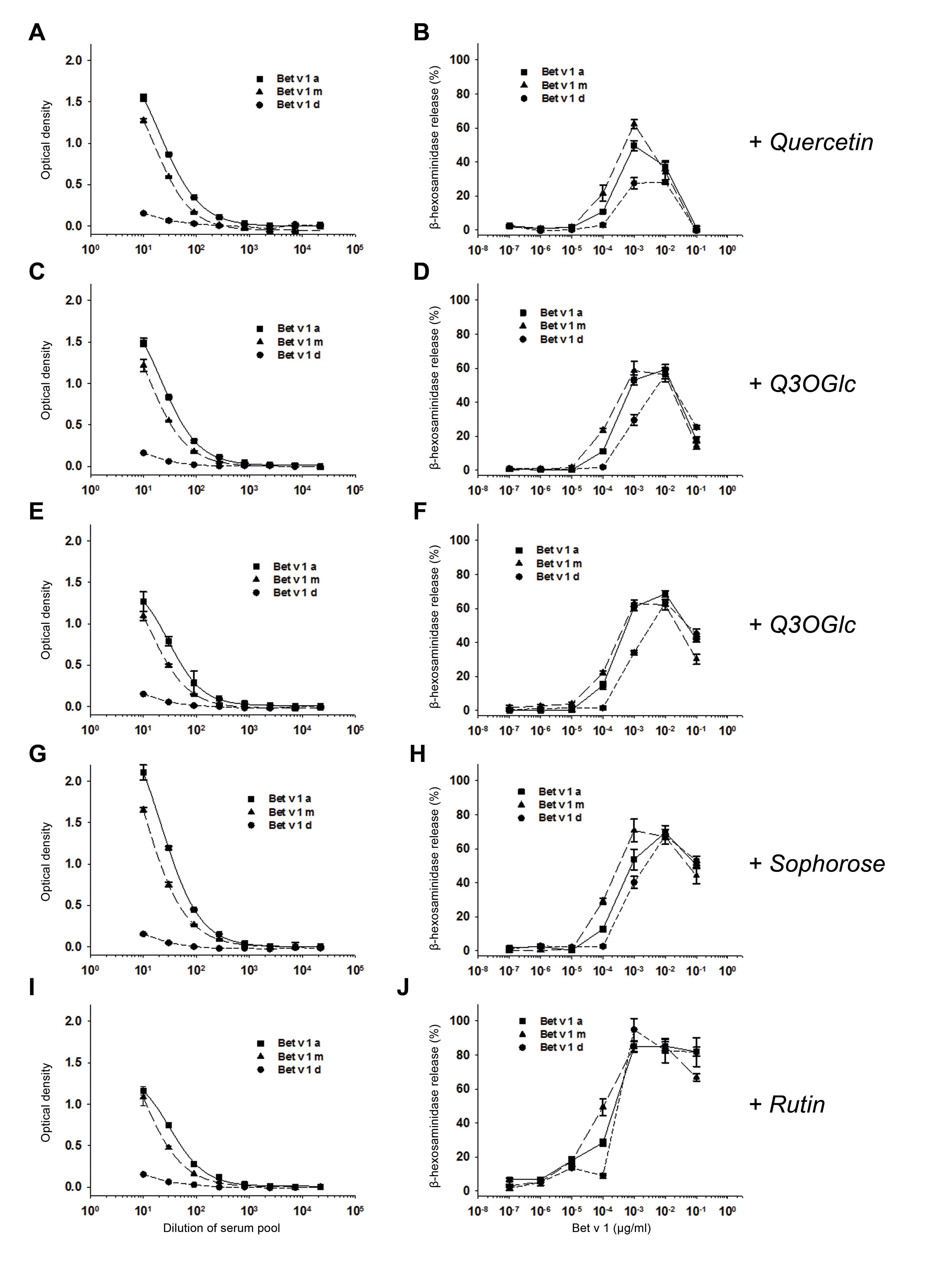

Supplement: S3 Fig — The left panel shows binding of serial dilutions of serum IgE to equimolar amounts of surface-coated Bet v 1a (■), Bet v 1d (●), and Bet v 1m (▲) with 5-molar excess of A quercetin, C Q3OGlc, E Q3OGal, G sophorose, and I rutin respectively. Mediator release induced by recombinant Bet v 1 isoforms is illustrated in the right panel. Humanized RBL cells were sensitized with a pool of human birch-specific sera. Cross-linking of membrane-bound human IgE by IgE-Bet v 1 isoform interaction and subsequent release of β-hexosaminidase was determined with serial dilutions of Bet v 1a (■), Bet v 1d (●), and Bet v 1m (▲) with 5-molar excess of B quercetin, D Q3OGlc, F Q3OGal, H sophorose, and J rutin respectively. (TIF) [file pone.0128677.s003.tif]

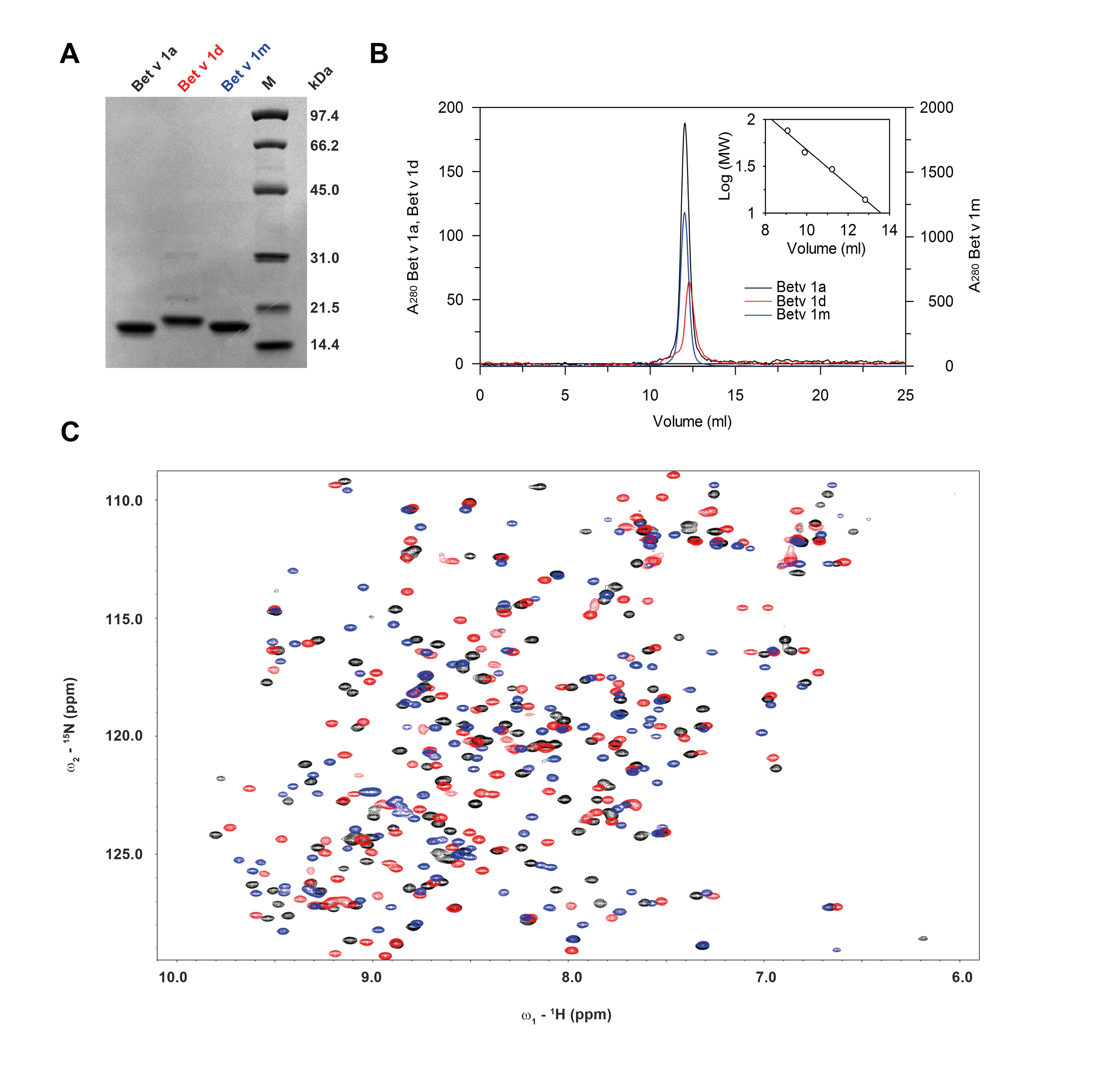

Supplement: S4 Fig — A SDS/PAGE on 19% gels of ca. 1 μg Bet v 1 isoforms (MW 17.4 kDa) after purification. M, molecular-mass standard (Low Range, Bio-Rad Laboratories). B SEC of the isoforms performed with a Superdex S75 GL 10/300 column (total bed volume: 24 ml; GE Healthcare) in 50 mM sodium phosphate, 50 mM NaCl, pH 7.0 at RT. Column calibration was performed with conalbumin (75.0 kDa), ovalbumin (43.0 kDa), carbonic anhydrase (29.0 kDa) and ribonuclease (13.7 kDa). The elution profile of 0.5 mg Bet v 1a is shown in black, 0.25 mg of Bet v 1d in red and 2.4 mg of Bet v 1m in blue. The peaks correspond to monomeric proteins with molecular masses of 19.66 kDa (Bet v 1a), 17.63 kDa (Bet v 1d) and 19.74 kDa (Bet v 1m). C 1H-15N HSQC spectra of 100 μM Bet v 1a (black), Bet v 1d (red) and Bet v 1m (blue) in 50 mM sodium phosphate, 50 mM NaCl, pH 7.0 and 10% 2H2O at 298 K. (TIF) [file pone.0128677.s004.tif]
