# Supplementary material for: Ligand Recognition of the Major Birch Pollen Allergen Bet v 1 is Isoform Dependent
Source: PLoS One. 2015 Jun 4;10(6):e0128677. doi: 10.1371/journal.pone.0128677 (PMC4456386; doi:10.1371/journal.pone.0128677)
Supplement: S1 Table — (DOCX) [file pone.0128677.s005.docx]

### Supporting Information

**S1 Table. Bet v 1a residues affected from addition of flavonoids with CSPs showing ∆δ_norm_ > 0.08 ppm.**

Flavonoids were stepwise added to a final excess of up to 17-fold to 100 µM of ^15^N-labelled Bet v 1a. The ∆δ_norm_-values were determined with equation 2. K_d_ values for flavonoid binding were determined with NMRViewJ [89].

n.a.: Data could not be analysed. Int. Ex.: Intermediate exchange

| **Flavone** | | | **Naringenine** | | | | **Fisetin** | | | | **Quercetin** | | | | | **Myricetin** | |
| --- | --- | --- | --- | --- | --- | --- | --- | --- | --- | --- | --- | --- | --- | --- | --- | --- | --- |
| Residue | *K*_d_ (*µ*M) | | Residue | | *K*_d_ (*µ*M) | | Residue | | | *K*_d_ (*µ*M) | Residue | | | *K*_d_ (*µ*M) | | Residue | *K*_d_ (*µ*M) |
| E6 | n.a. | | T10 | | 11.5±3.2 | | T10 | | | 36.8±7.1 | T10 | | | 36.8±7.1 | | T10 | Int. Ex. |
| T7 | 140.3±41.6 | | A15 | | 7.3±4.0 | | S11 | | | 18.6±7.6 | A15 | | | 11.1±8.3 | | A15 | 11.6±5.5 |
| T10 | 118.5±5.6 | | F22 | | 80.3±20.6 | | A15 | | | 28.6±2.6 | G89 | | | 21.6±3.6 | | G26 | Int. Ex. |
| A15 | 19.0±4.9 | | G26 | | n.a. | | G26 | | | n.a. | K115 | | | 18.6±4.3 | | I38 | Int. Ex. |
| L24 | 65.1±7.9 | | F64 | | 10.4±4.2 | | G89 | | | 17.4±3.8 | K137 | | | Int. Ex. | | S39 | 26.9±13.3 |
| G26 | 64.4±11.3 | | K68 | | 59.3±0.9 | | I91 | | | 16.8±5.7 | E138 | | | Int. Ex. | | E73 | Int. Ex. |
| A27 | 57.9±13.8 | | R70 | | 12.5±5.1 | | I102 | | | 18.2±3.7 | G140 | | | Int. Ex. | | Y81 | Int. Ex. |
| F30 | n.a. | | Y83 | | 12.0±5.3 | | K115 | | | 39.4±6.7 | R145 | | | 69.1±18.8 | | N82 | Int. Ex. |
| A34 | 41.2±2.8 | | G89 | | 32.5±4.2 | | S117 | | | 72.5±15.4 |  | | |  | | G89 | 5.0±2.2 |
| F64 | 5.9±1.1 | | I102 | | 39.5±8.5 | | S136 | | | 16.1±3.8 |  | | |  | | K115 | Int. Ex. |
| G89 | 21.6±3.6 | | K115 | | 30.7±8.6 | | K137 | | | n.a. |  | | |  | | S136 | Int. Ex. |
| I102 | 29.9±3.8 | | S117 | | n.a. | | E138 | | | 53.5±19.3 |  | | |  | | K137 | Int. Ex. |
| K115 | 143.1±39.3 | | K137 | | 12.9±3.2 | | G140 | | | Int. Ex. |  | | |  | | E138 | Int. Ex. |
| S117 | 60.5±11.8 | | E138 | | 32.6±15.5 | | L143 | | | n.a. |  | | |  | | G140 | Int. Ex. |
| V128 | n.a. | | G140 | | Int. Ex. | | L144 | | | n.a. |  | | |  | | T142 | n.d |
| V133 | 47.2±8.4 | | E141 | | n.a. | | R145 | | | 48.0±8.6 |  | | |  | | L143 | n.d |
| S136 | n.a. | | T142 | | n.d | |  | | |  |  | | |  | | R145 | n.a. |
| E138 | n.a. | | L143 | | n.d | |  | | |  |  | | |  | |  |  |
| M139 | n.a. | | L144 | | n.a. | |  | | |  |  | | |  | |  |  |
| E141 | 142.5±38.0 | | R145 | | n.d | |  | | |  |  | | |  | |  |  |
| T142 | n.a. | |  | |  | |  | | |  |  | | |  | |  |  |
|  |  | |  | |  | |  | | |  | |  | | |  |  |  |
| **Q3OGlc** | | | | **Q3OGal** | | | | **Q3OS** | | | | |  |  |  |  |  |
| Residue | | *K*_d_ (*µ*M) | | Residue | | *K*_d_ (*µ*M) | | Residue | *K*_d_ (*µ*M) | | | |  |  |  |  |  |
| T10 | | 357.3±24.0 | | F22 | | Int. Ex. | | F22 | Int./Slow Ex. | | | |  |  |  |  |  |
| G26 | | 259.2±5.6 | | I23 | | Int. Ex. | | L29 | Int./Slow Ex. | | | |  |  |  |  |  |
| G89 | | 285.0±30.2 | | G26 | | Int. Ex. | | I38 | Int./Slow Ex. | | | |  |  |  |  |  |
| K137 | | Int. Ex. | | K54 | | Int. Ex. | | K55 | Int./Slow Ex. | | | |  |  |  |  |  |
| E138 | | Int. Ex. | | F64 | | Int. Ex. | | R70 | Int./Slow Ex. | | | |  |  |  |  |  |
| G140 | | Int. Ex. | | R70 | | Int. Ex. | | E73 | Int./Slow Ex. | | | |  |  |  |  |  |
| T142 | | 252.1±42.0 | | E73 | | Int. Ex. | | V74 | Int./Slow Ex. | | | |  |  |  |  |  |
|  | |  | | G92 | | Int. Ex. | | N82 | Int./Slow Ex. | | | |  |  |  |  |  |
|  | |  | | D93 | | Int. Ex. | | S84 | Int./Slow Ex. | | | |  |  |  |  |  |
|  | |  | | S136 | | Int. Ex. | | V85 | Int./Slow Ex. | | | |  |  |  |  |  |
|  | |  | | K137 | | Int. Ex. | | K115 | Int./Slow Ex. | | | |  |  |  |  |  |
|  | |  | | E138 | | Int. Ex. | | Y120 | Int./Slow Ex. | | | |  |  |  |  |  |
|  | |  | | G140 | | Int. Ex. | | K137 | Int./Slow Ex. | | | |  |  |  |  |  |
|  | |  | | E141 | | Int. Ex. | | E138 | Int./Slow Ex. | | | |  |  |  |  |  |
|  | |  | | T142 | | n.a. | | G140 | Int./Slow Ex. | | | |  |  |  |  |  |
|  | |  | | L144 | | Int. Ex. | | L144 | Int./Slow Ex. | | | |  |  |  |  |  |
|  | |  | | V147 | | n.a. | |  |  | | | |  |  |  |  |  |
