# Supplementary material for: Ligand Recognition of the Major Birch Pollen Allergen Bet v 1 is Isoform Dependent
Source: PLoS One. 2015 Jun 4;10(6):e0128677. doi: 10.1371/journal.pone.0128677 (PMC4456386; doi:10.1371/journal.pone.0128677)
Supplement: S2 Table — (DOCX) [file pone.0128677.s006.docx]

### Supporting Information

**S2 Table**. **Bet v 1m residues affected from addition of flavonoids with CSPs showing ∆δ_norm_ > 0.08 ppm.**

Flavonoids were stepwise added to a final excess of up to 17-fold to 100 µM of ^15^N-labelled Bet v 1m. The ∆δ_norm_-values were determined with equation 2. K_d_ values for flavonoid binding were determined with NMRViewJ [89].

n.a.: Data could not be analysed. Int. Ex.: Intermediate exchange

| **Flavone** | | **Naringenine** | | | **Fisetin** | | | **Quercetin** | | **Myricetin** | |
| --- | --- | --- | --- | --- | --- | --- | --- | --- | --- | --- | --- |
| Residue | *K*_d_ (*µ*M) | Residue | *K*_d_ (*µ*M) | | Residue | *K*_d_ (*µ*M) | | Residue | *K*_d_ (*µ*M) | Residue | *K*_d_ (*µ*M) |
| S7 | 556.3±152.7 | G26 | 19.0±5.3 | | I23 | Int. Ex. | | E6 | n.a. | V41 | 88.5±14.5 |
| T9 | 467.8±152.8 | T57 | n.a. | | T57 | 110.6±29.9 | | I23 | n.a. | I56 | 126.0±23 |
| I23 | 16.3±6.3 | A90 | Int. Ex. | | G88 | 81.3±33.5 | | G26 | 47. ±9.1 | T57 | Int. Ex. |
| G26 | 128.6±12.3 | N118 | 39.6±9.7 | | G89 | n.a. | | V33 | n.a. | Y66 | 120.3±42 |
| D27 | 103.8±14.0 | K137 | 27.1±3.4 | | A90 | Int. Ex. | | A34 | n.a. | M85 | n.a. |
| A34 | 72.2±10.0 | E138 | Int. Ex. | | I102 | 20.4±9.3 | | S39 | n.a. | G89 | Int. Ex. |
| A37 | 265.1±19.4 | K139 | 8.2±5.0 | | S117 | n.a. | | S40 | n.a. | A90 | Int. Ex. |
| S39 | 436.1±201.4 | G140 | 13.6±7.2 | | K137 | 149.7±47.4 | | V41 | 72.7±21.2 | G92 | Int. Ex. |
| S40 | 356.0±91.8 | E141 | n.a. | | E138 | Int. Ex. | | I56 | n.a. | S117 | 75.3±10.6 |
| V41 | 117.8±24.3 |  |  | | K139 | 28.9±3.4 | | T57 | Int.Ex. | K134 | 161.4±13 |
| I56 | 195.0±29.1 |  |  | | G140 | Int. Ex. | | F64 | 40.5 | E138 | 24.4±13.2 |
| T57 | n.a. |  |  | | A142 | 109.4±27.6 | | Y66 | n.a. | K139 | Int. Ex. |
| Y66 | 125.9±16.4 |  |  | | L143 | Int. Ex. | | E73 | n.a. | E141 | Int. Ex. |
| Y81 | 116±7.9 |  |  | |  |  | | S84 | Int. Ex. | L143 | Int. Ex. |
| I98 | 419.1±81.6 |  |  | |  |  | | M85 | Int. Ex. | L144 | Int. Ex. |
| N100 | 24.6±6.9 |  |  | |  |  | | G89 | n.a. |  |  |
| E138 | 190.9±45.1 |  |  | |  |  | | L91 | 90.4±37.6 |  |  |
| K139 | 301.1±84.1 |  |  | |  |  | | N100 | Int. Ex. |  |  |
| E141 | 45.0±10.7 |  |  | |  |  | | I102 | n.a. |  |  |
| L144 | 287.9±64.5 |  |  | |  |  | | N118 | 21.4±8.9 |  |  |
| S149 | 40.1±6.0 |  |  | |  |  | | K134 | 45.3±3.1 |  |  |
|  |  |  |  | |  |  | | K137 | Int. Ex. |  |  |
|  |  |  |  | |  |  | | E138 | Int. Ex. |  |  |
|  |  |  |  | |  |  | | K139 | 57.2±9.5 |  |  |
|  |  |  |  | |  |  | | G140 | n.a. |  |  |
|  |  |  |  | |  |  | | E141 | 11.6±7.5 |  |  |
|  |  |  |  | |  |  | | A142 | 118.5±2.9 |  |  |
|  |  |  |  | |  |  | | L144 | n.a. |  |  |
|  |  |  |  | |  |  | |  |  |  |  |
| **Q3OGlc** | | **Q3OGal** | | | **Q3OS** | | |  |  |  |  |
| Residue | *K*_d_ (*µ*M) | Residue | | *K*_d_ (*µ*M) | Residue | | *K*_d_ (*µ*M) |  |  |  |  |
| E6 | Int. Ex. | I38 | | Int. Ex. |  | |  |  |  |  |  |
| I23 | Int. Ex. | S39 | | Int. Ex. |  | |  |  |  |  |  |
| G26 | Int. Ex. | T57 | | Int. Ex. |  | |  |  |  |  |  |
| I38 | Int. Ex. | F64 | | Int. Ex. |  | |  |  |  |  |  |
| T57 | Int. Ex. | Y66 | | Int. Ex. |  | |  |  |  |  |  |
| F64 | Int. Ex. | M85 | | Int. Ex. |  | |  |  |  |  |  |
| Y66 | Int. Ex. | I86 | | Int. Ex. |  | |  |  |  |  |  |
| G89 | Int. Ex. | A90 | | Int. Ex. |  | |  |  |  |  |  |
| A90 | Int. Ex. | L91 | | Int. Ex. |  | |  |  |  |  |  |
| L91 | Int. Ex. | G92 | | Int. Ex. |  | |  |  |  |  |  |
| S117 | Int. Ex. | E96 | | Int. Ex. |  | |  |  |  |  |  |
| N118 | Int. Ex. | Y120 | | Int. Ex. |  | |  |  |  |  |  |
| I136 | Int. Ex. | K134 | | Int. Ex. |  | |  |  |  |  |  |
| K137 | Int. Ex. | I136 | | Int. Ex. |  | |  |  |  |  |  |
| E138 | Int. Ex. | K137 | | Int. Ex. |  | |  |  |  |  |  |
| K139 | Int. Ex. | E138 | | Int. Ex. |  | |  |  |  |  |  |
| G140 | Int. Ex. | K139 | | Int. Ex. |  | |  |  |  |  |  |
| E141 | Int. Ex. | G140 | | Int. Ex. |  | |  |  |  |  |  |
| A142 | Int. Ex. | E141 | | Int. Ex. |  | |  |  |  |  |  |
| L143 | Int. Ex. | L143 | | Int. Ex. |  | |  |  |  |  |  |
| L144 | Int. Ex. | L144 | | Int. Ex. |  | |  |  |  |  |  |
