# Supplementary material for: Ligand Recognition of the Major Birch Pollen Allergen Bet v 1 is Isoform Dependent
Source: PLoS One. 2015 Jun 4;10(6):e0128677. doi: 10.1371/journal.pone.0128677 (PMC4456386; doi:10.1371/journal.pone.0128677)
Supplement: S3 Table — (DOCX) [file pone.0128677.s007.docx]

### Supporting Information

**S3 Table**. **Bet v 1d residues affected from addition of flavonoids with CSPs showing ∆δ_norm_ > 0.08 ppm.**

Flavonoids were stepwise added to a final excess of up to 17-fold to 100 µM of ^15^N-labelled Bet v 1d. The ∆δ_norm_-values were determined with equation 2. K_d_ values for flavonoid binding were determined with NMRViewJ [89].

n.a.: Data could not be analysed.

| **Flavone** | | **Q3OGal** | | | | **Q3OS** | | | |  |  |
| --- | --- | --- | --- | --- | --- | --- | --- | --- | --- | --- | --- |
| Residue | *K*_d_ (*µ*M) | Residue | | *K*_d_ (*µ*M) | | Residue | | *K*_d_ (*µ*M) | |  |  |
| E6 | 98.2±7.2 | Q132 | | n.a. | | F64 | | n.a. | |  |  |
| T9 | 73.2±5.7 | S136 | | n.a. | | A135 | | n.a. | |  |  |
| A21 | 12.6±6.3 |  | |  | | S136 | | n.a. | |  |  |
| L24 | 42.8±12.9 |  | |  | |  | |  | |  |  |
| D25 | 85.0±21.8 |  | |  | |  | |  | |  |  |
| D27 | 18.8±7.1 |  | |  | |  | |  | |  |  |
| N28 | n.a. |  | |  | |  | |  | |  |  |
| V30 | 45.7±15.1 |  | |  | |  | |  | |  |  |
| K32 | n.a. |  | |  | |  | |  | |  |  |
| Q36 | n.a. |  | |  | |  | |  | |  |  |
| A37 | n.a. |  | |  | |  | |  | |  |  |
| I38 | 43.1±1.5 |  | |  | |  | |  | |  |  |
| K55 | 24.8±10.5 |  | |  | |  | |  | |  |  |
| Y66 | 183.3±44.6 |  | |  | |  | |  | |  |  |
| G88 | 14.5±5.1 |  | |  | |  | |  | |  |  |
| V91 | n.a. |  | |  | |  | |  | |  |  |
| G92 | 45.7±15.1 |  | |  | |  | |  | |  |  |
| E96 | 65.1±1.3 |  | |  | |  | |  | |  |  |
| E101 | n.a. |  | |  | |  | |  | |  |  |
| K103 | 14.1±2.3 |  | |  | |  | |  | |  |  |
| N125 | 52.4±9.0 |  | |  | |  | |  | |  |  |
| V128 | 183.5±13.5 |  | |  | |  | |  | |  |  |
| K129 | 256.1±30.2 |  | |  | |  | |  | |  |  |
| V133 | 72.3±6.2 |  | |  | |  | |  | |  |  |
| G140 | n.a. |  | |  | |  | |  | |  |  |
| L144 | 33.1±4.8 |  | |  | |  | |  | |  |  |
|  |  |  |  | |  | |  | |  | |  |
