# Supplementary material for: Ligand Recognition of the Major Birch Pollen Allergen Bet v 1 is Isoform Dependent
Source: PLoS One. 2015 Jun 4;10(6):e0128677. doi: 10.1371/journal.pone.0128677 (PMC4456386; doi:10.1371/journal.pone.0128677)
Supplement: S4 Table — (DOCX) [file pone.0128677.s008.docx]

### Supporting Information

**S4 Table. Absorption maxima of unglycosylated flavonoids and their Bet v 1-complexes.**

Due to overlay of the flavone absorption maxima with the protein absorption, the maxima of the corresponding Bet v 1-complexes could not be identified with certainty (n.a.). Not measured (-)

|  | **Absorption maxima (nm)** | | | |
| --- | --- | --- | --- | --- |
|  | **Flavonoid** | **Bet v 1a:Flavonoid** | **Bet v 1m:Flavonoid** | **Bet v 1d:Flavonoid** |
| Flavone | 302 | n.a. | n.a. | - |
| Naringenin | 325 | 325 | 322 | 325 |
| Fisetin | 368 | 372 | 370 | 371 |
| Quercetin | 377 | 382 | 381 | 382 |
| Myricetin | 378 | 387 | n.a. | 382 |
